# A Four-Hour Carbapenem Inactivation Method (CIM^**B.S**^) Using *Bacillus stearothermophilus* as Indicator Strain

**DOI:** 10.3389/fmed.2020.00364

**Published:** 2020-07-31

**Authors:** Ze-Hua Cui, Ling Jia, Lu Han, Tian Tang, Zi-Xing Zhong, Liang-Xing Fang, Wei-Na Ni, Min-Ge Wang, Xi-Ran Wang, Ya-Hong Liu, Xiao-Ping Liao, Jian Sun

**Affiliations:** ^1^National Risk Assessment Laboratory for Antimicrobial Resistance of Animal Original Bacteria, South China Agricultural University, Guangzhou, China; ^2^Laboratory of Veterinary Pharmacology, College of Veterinary Medicine, South China Agricultural University, Guangzhou, China; ^3^Guangdong Laboratory for Lingnan Modern Agriculture, Guangzhou, China

**Keywords:** *Bacillus stearothermophilus*, carbapenem inactivation method, rapid, colorimetric, phenotype detection

## Abstract

**Objectives:** There is an urgent need for accurate and fast diagnostic tests to identify carbapenemase-producing bacteria. Here we used *Bacillus stearothermophilus* as an indicator strain in the format of the carbapenem inactivation method (CIM) procedure to develop a rapid carbapenemase phenotype detection method: CIM^B.S^.

**Methods:** The CIM^B.S^ test was derived from the mCIM, where *B. stearothermophilus* replaced *Escherichia coli* as the indicator strain. The test bacteria were incubated in the presence of imipenem for 30 min, and then, aliquots were placed on colorimetric plates, and incubation was continued for 3.5 h at 60°C. We examined 134 clinical strains to evaluate the CIM^B.S^ performance.

**Results:** The CIM^B.S^ can be completed in 4 h, and we successfully identified 38/39 (97.4%) carbapenemase-producing Enterobacteriaceae, including 17/18 (94.4%) carbapenemase-producing *Pseudomonas aeruginosa* and 18/19 (94.7%) carbapenemase-producing *Acinetobacter baumannii*. All non-carbapenemase producers we tested were negative and included Enterobacteriaceae (*n* = 36), *P. aeruginosa* (*n* = 17), and *A. baumannii* (*n* = 5).

**Conclusions:** The CIM^B.S^ test is a rapid carbapenemase phenotype detection method requiring only 4 h of total work time and displays high sensitivity and specificity.

## Introduction

The carbapenems are a highly efficacious group of antibiotics and are considered as the last resort for treating multidrug-resistant Enterobacteriaceae (1). However, infections caused by carbapenem-resistant Gram-negative bacteria (CRGB) have been increasing (2). The global spread of CRGB is an urgent public health challenge due to limited therapeutic options, such that these infections are often accompanied by high mortality rates (3). Carbapenem resistance is due to decreased porin expression, increased expression of multidrug efflux pumps, and overexpression of AmpC and carbapenemases. The latter is the primary resistance mechanism for these infections (4).

Carbapenemase genes are frequently located on mobile genetic elements, including plasmids, and this increases transfer frequencies to other bacteria (5, 6). Thus, a crucial element to prevent dissemination of carbapenemase resistance is early detection of CRGB (7). Unfortunately, the classic methods of antibiotic susceptibility testing are not completely effective for CRGB because many are not resistant to meropenem, such as the partial OXA-48-producing strains (8). In addition, molecular techniques, such as PCR and whole genome sequencing, are available but rely on expensive equipment and supplies (9). Phenotype detection is an important adjunct to molecular detection, such as the Carba NP test and matrix-assisted laser desorption/ionization time-of-flight mass spectrometry, which detects carbapenemase hydrolysis products (10), as well as lateral flow immunoassays that rely on anti-carbapenemase antibodies (11).

In contrast to these assays, the modified Hodge test (MHT) and modified carbapenem inactivation (mCIM) methods are growth-based assays that determine the extent of antibiotic resistance based on the growth of an indicator organism in the presence of the antibiotic (12, 13). The mCIM is widely used in clinical laboratories because of its simplicity, accuracy, and low cost, but the procedure is time-consuming (13). However, these time constraints can be overcome by use of carbapenemase-susceptible bacteria that can grow rapidly. For example, *Bacillus stearothermophilus* is a Gram-positive thermophile that grows at 45–75°C and is an acid producer that can rapidly alter the pH of the growth medium (14). Here, we used *B. stearothermophilus* as indicator strain to develop the CIM^B.S^, a rapid carbapenemase phenotype detection method derived from mCIM that reduces the carbapenemase phenotype detection time from more than 18–4 h.

## Materials and Methods

### Strains and Reagents

We used 134 clinical isolates that were provided by Huizhou First People's Hospital and The Third Affiliated Hospital of Sun Yat-sen University (Table S1). All tested strains were identified to the species level by using MALDI-TOF MS (Axima-Assurance, Shimadzu, Kyoto, Japan). These clinical strains were used to evaluate CIM^B.S^ test performance and included 76 isolates that possessed the carbapenemase genes NDM, KPC, VIM, IMP, or OXA and 58 strains that did not carry any carbapenemase gene. This strain collection was composed of 39 carbapenemase-producing Enterobacteriaceae (CPE) that included 14 *E. coli* (NDM-1, NDM-5, VIM-2, and IMP-2), 16 *Klebsiella pneumoniae* (NDM-1, NDM-5, VIM-2, IMP-2, and KPC-2), 2 *Citrobacter freundii* (NDM-1), and 7 *Enterobacter cloacae* (NDM-1, VIM-1, and IMP-2) as well as 18 carbapenemase-producing *Pseudomonas aeruginosa* (CPPA) (NDM-5, VIM-2, and IMP-2) and 19 carbapenemase-producing *Acinetobacter baumannii* (CPAB) isolates (NDM-1, OXA-23, and KPC-2). The 58 non-carbapenemase-producing strains included 18 *E. coli*, 6 *K. pneumoniae*, 8 *C. freundii*, and 4 *E. cloacae* as well as 17 *P. aeruginosa* and 5 *A. baumannii*.

Ertapenem, meropenem, imipenem, bromothymol blue, bromocresol purple, and ZnSO_4_ were obtained from Sigma (St. Louis, MO, USA). *B. stearothermophilus* ATCC 7453 and *E. coli* strain ATCC 25922 were obtained from the American Type Culture Collection (Manassas, VA, USA). Imipenem disks and Mueller-Hinton agar were obtained from Oxoid (Bassingstoke, UK).

Carbapenemase genes were identified using PCR, and the following genes were included: *bla*_KPC_, *bla*_NDM_, *bla*_IMP_, or *bla*_VIM_ (15). PCR amplicons were sequenced using Sanger sequencing.

### Antibiotic Susceptibility Testing

Standard antimicrobial susceptibility assays were performed and interpreted according to the CLSI guidelines (16) using the agar dilution method with ertapenem, meropenem, and imipenem as the test agents and ATCC 25922 as the quality control strain.

### Carbapenemase Activity Assays

The Blue-Carba assay was performed as previously described (17). Briefly, a loop of a pure bacterial culture was suspended in 100 μL of both test and negative-control solution in a 96-well-microtiter plate and incubated at 37°C with agitation (150 rpm) for 2 h. The test solution consisted of 0.04% bromothymol blue at pH 6.0 containing ZnSO_4_ (0.1 mM) and imipenem (3 mg/mL) at a final pH of 7. The negative-control solution consisted of 0.04% bromothymol blue pH 7. If the test isolates produced carbapenemase, the color of the wells turned from blue to yellow; otherwise, the test isolate was considered negative.

The mCIM was performed as previously described with slight modifications (13). Briefly, a loopful (1 μL for Enterobacteriaceae and 10 μL for *P. aeruginosa and A. baumannii*) was added into 400 μL of sterile water containing a 10-μg imipenem disk and incubated for 30 min. The disk was then placed on a Mueller-Hinton agar plate containing the susceptible *E. coli* indicator strain ATCC 29522 and incubated for 18 h. Carbapenemase production by the test strain was indicated by growth of the indicator bacterium.

Colorimetric plates used for CIM^B.S^ assays were prepared using a filter-sterilized solution of 18 mg bromocresol purple/10 mL dH_2_O water that was combined with 10 mL of exponential phase culture (1 × 10^8^ cfu/mL) of *B. stearothermophilus* 7453 that was added to 980 mL freshly prepared and cooled (45–50°C) MH agar (pH 7.0). The plates were prepared using 20 mL in 90-mm bacteriological petri dishes to obtain a 4-mm depth or an equivalent to 70 mL in 150 mm dishes. The plates were stored at 4°C and were discarded after 7 days if not used for testing.

**Figure 1:**
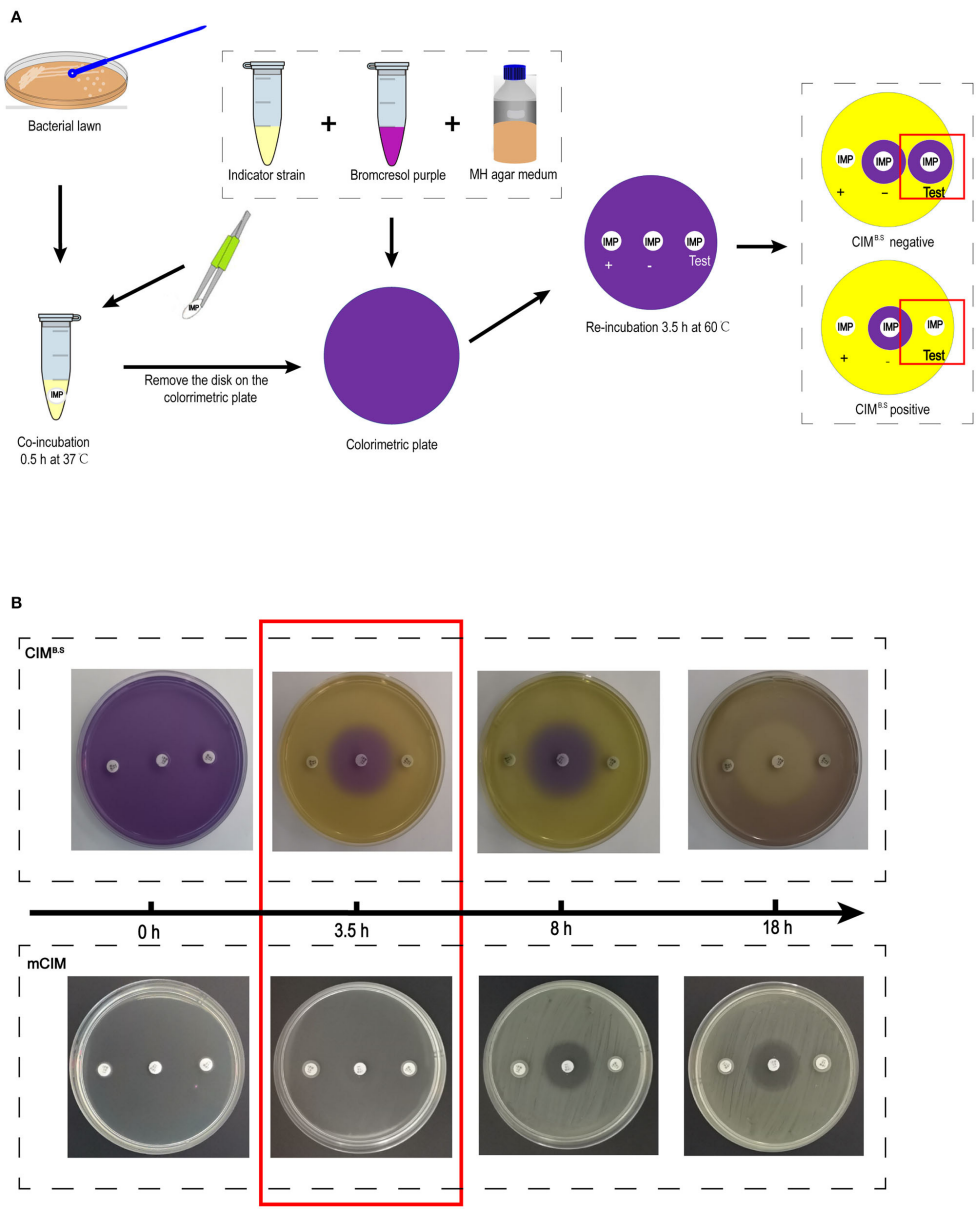
Identification of carbapenemase-producing bacteria using the CIM^B.S^ test. **(A)** Strategy of the test. The results are determined by the size of the colorimetric inhibition zone as compared with negative control. **(B)** Comparison of re-incubation time between the CIM^B.S^ and mCIM. The re-incubation time of CIM^B.S^ required only 3.5 h, which is shortened by 4.5 h from mCIM.

The CIM^B.S^ testing procedure was derived from the mCIM. One loopful (1 μL for Enterobacteriaceae and 10 μL for *P. aeruginosa and A. baumannii*) of overnight liquid cultures was added to Eppendorf tubes containing 400 μL sterile water. One 10-μg imipenem disk was added to the suspension that was vortexed for 1 min and incubated for 30 min at 37°C. The disk was then placed on a colorimetric plate and incubated for 3.5 h at 60°C (Figure 1A).

Acid production by the indicator strain results in a color change from purple (pH 7) to yellow (pH < 5.8) resulting in colored zones that were measured with a Vernier caliper. If the tested strain produced carbapenemase, imipenem is degraded, allowing the growth of the susceptible indicator strain and a yellow colorimetric zone.

### Statistical Analysis

The sensitivity was calculated by dividing the number of positive strains by the number of strains harboring the carbapenenase genes. The specificity was calculated by dividing the number of strains with negative results by the number of strains without the carbapenem gene. All experiments were performed in triplicate. Statistical analyses were performed using GraphPad Prism 7 (GraphPad Software) and Excel 2010 (Microsoft, Redmond, WA, USA).

## Results

### Detection Time of the CIM^B.S^

The CIM^B.S^ was evaluated on 134 tested strains to detect a carbapenemase activity, and it can finish the carbapenemase phenotypic detection in 4 h by use of *B. stearothermophilus* as indicator strain with result same as mCIM (Figure S1).

After incubation with the test bacteria culture, the imipenem disk was placed on a colorimetric plate at 60°C, and colorimetric zones started to form and became clearly visible by 3.5 h (Figure 1B). This incubation time can be extended appropriately, but prolonged incubation should be avoided because further medium acidification blurred at 8 h, and the colorimetric zones in the positive control disappeared at ~18 h. However, at 18 h, inhibition zones similarly to those produced in the mCIM assay can be used for interpretation.

### Sensitivity and Specificity of CIM^B.S^

We also examined the specificity of our CIM^B.S^ using known carbapenemase producers. In our group of Enterobacteriaceae, we successfully identified 38/39 that were CIM^B.S^ positive and one *E. coli* strain carrying the NDM-5 gene that was negative. All 36 non-CPE were CIM^B.S^ negative. Overall, the sensitivity and specificity of the CIM^B.S^ for the Enterobacteriaceae were 97.4 and 100%, respectively (Table 1).

**Table 1:** Carbapenemase and non-carbapenemase-producing Enterobacteriaceae tested.

			**MIC (mg/L)**			**No. of isolates with a positive test/no. of isolates tested**		
**Species**	***n***	**Carbapenemase**	**Ertapenem**	**Imipenem**	**Meropenem**	**Blue-Carba**	**mCIM**	**CIM^**B.S**^**
Test strains	75							
carbapenemase-producers	39							
*E. coli*	3	NDM-1	4– >64	2->64	16–>64	3/3	3/3	3/3
*E. coli*	8	NDM-5	8–>64	2–>64	2–>64	6/8	7/8	7/8
*E. coli*	2	VIM-2	>64	>64	>64	2/2	2/2	2/2
*E. coli*	1	IMP-2	>64	32	32	1/1	1/1	1/1
*K. pneumoniae*	3	NDM-1	16–64	4–32	4–32	3/3	3/3	3/3
*K. pneumoniae*	3	NDM-5	≥64	16–64	16–64	3/3	3/3	3/3
*K. pneumoniae*	1	VIM-2	64	32	32	1/1	1/1	1/1
*K. pneumoniae*	1	IMP-2	>64	64	16	1/1	1/1	1/1
*K. pneumoniae*	8	KPC-2	16–≥64	16–64	4–>64	8/8	8/8	8/8
*C. freundii*	2	NDM-1	64	8–16	8–16	2/2	2/2	2/2
*E. cloacae*	3	NDM-1	32–64	4–16	4–16	3/3	3/3	3/3
*E. cloacae*	1	VIM-1	4	4	2	1/1	1/1	1/1
*E. cloacae*	3	IMP-2	8–64	2–8	2–16	3/3	3/3	3/3
Non-carbapenemase-producers	36							
*E. coli*	18	-	0.125–64	≤ 0.125–32	<0.125–16	0/18	0/18	0/18
*K. pneumoniae*	6	-	0.125–16	≤ 0.125–8	<0.125–8	0/6	0/6	0/6
*C. freundii*	8	-	<0.125–0.5	<0.125–0.5	<0.125	0/8	0/8	0/8
*E. cloacae*	4	-	2–8	0.25–0.5	<0.125	0/4	0/4	0/4

*n, number of isolates tested*.

When we assayed the *P. aeruginosa* strains, 18/18 CPPA were CIM^B.S^ positive, and all 17 non-CPPA isolates tested CIM^B.S^ negative. The corresponding sensitivity and specificity were 100% (Table 2). In the *A. baumannii* group, we found that 18/19 of the CPAB were positive, and one CPAB strain carrying the NDM-5 gene that was negative; all 5 non-CPAB isolates were CIM^B.S^ negative. The sensitivity and specificity of the CIM^B.S^ for the *A. baumannii* were 94.7 and 100%, respectively (Table 2).

**Table 2:** Carbapenemase and non-carbapenemase-producing *P. aeruginosa A. baumannii* and tested.

			**MIC (mg/L)**			**No. of isolates with a positive test/no. of isolates tested**		
**Species**	***n***	**Carbapenemase**	**Ertapenem**	**Imipenem**	**Species**	**Blue-Carba**	**Mcim**	**CIM^**B.S**^**
Test strains	35							
carbapenemase-producers	18							
*P. aeruginosa*	9	NDM-5	>64	32	32	7/9	9/9	9/9
*P. aeruginosa*	4	VIM-2	>64	>64	>64	4/4	4/4	4/4
*P. aeruginosa*	5	IMP-2	16–>64	8–>64	>64	5/5	5/5	5/5
*A. baumannii*	1	NDM-1	≥64	32–≥64	32–64	1/1	1/1	1/1
*A. baumannii*	17	OXA-23	≥64	32–≥64	32–64	3/17	16/17	16/17
*A. baumannii*	1	KPC-2	>64	>64	64	0/1	1/1	1/1
Non-carbapenemase-producers								
*P. aeruginosa*	17	–	8–32	2–32	2–32	0/17	0/17	0/17
*A. baumannii*	5	–	1–8	0.125–1	2–8	0/5	0/5	0/5

*n, number of isolates tested*.

**Figure 2:**
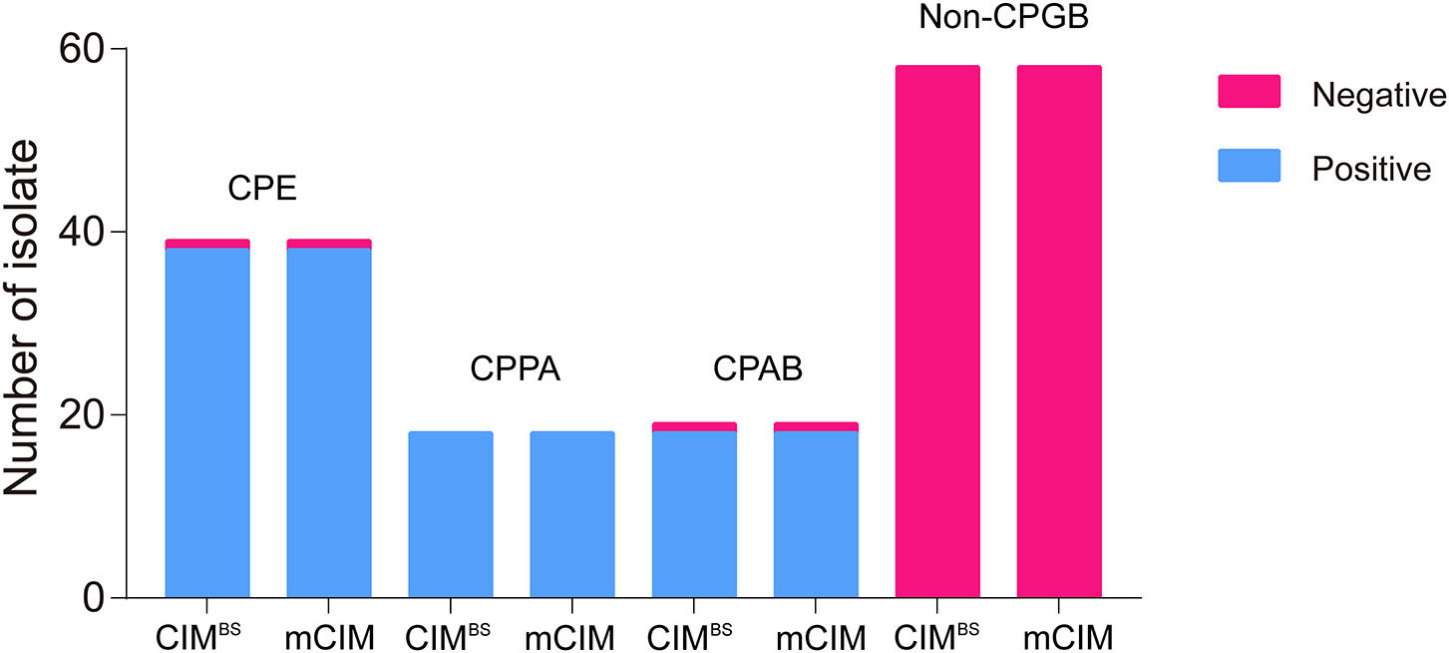
A comparison of test results for 128 bacterial strains using the CIM^B.S^ and mCIM assays. This strain collection was composed of 70 CPGB (39 CPE, 18 CPPA, and 19 CPAB) and 58 non-CPGB. Compared with the results of CIM^B.S^ and mCIM, there is no difference in detecting Enterobacteriaceae*, P. aeruginosa*, and *A. baumannii*.

All test results of CIM^B.S^ are consistent with mCIM, illustrating that the indicator strain changed from *E. coli* to *B. stearothermophilus* without affecting the sensitivity and specificity (Figure 2). However, compared with Blue-Carba, the results of CIM^B.S^ are different. A total of 18 tested strains (1 *E. coli*, 2 *P. aeruginosa*, and 14 *A. baumannii*) were Blue-Carba negative but CIM^B.S^ positive. In this study, when detecting Enterobacteriaceae and *P. aeruginosa*, the CIM^B.S^ and Blue-Carba are both highly sensitive; at the same time, CIM^B.S^ is more accurate. For detection of the *A. baumannii*, Blue-Carba is poorly sensitive (21.1%); however, the sensitivity of the CIM^B.S^ (94.1%) is much higher than Blue-Carba.

## Discussions and Conclusion

In this study, we presented the CIM^B.S^, which can rapidly detect carbapenemase activity in Enterobacteriaceae and *P. aeruginosa*. CIM^B.S^ is derived from mCIM, and there is no difference with the results, requiring only 4 h of total work time, which is compatible with the daily practice of a clinical microbiology laboratory.

Compared to the other carbapenemase activity detection method, CIM^B.S^ considerably shortens the time needed to identify the carbapenemase phenotype, such as the several days needed for whole-genome sequencing (18). Additionally, our assay does not require highly trained personnel or special equipment. Nonetheless, tests based on Carba-NP, such as the CIM^B.S^, require subjective interpretation (19). The mCIM is a carbapenemase activity detection method recommended by the CLSI and is widely used in clinical microbiology laboratories. The amount of time needed for this test is still the largest shortcoming, and our use of the *B. stearothermophilus* strain replacing *E. coli* provided a same-day result. A previous study changing the indicator strain from *E. coli* to *P. aeruginosa* PAO1 improved the accuracy of MHT but was still time-consuming (20). *B. stearothermophilus* is widely used in commercial enzyme production and as a biological sterilization indicator to detect antibiotic residues. *B. stearothermophilus* has also been used as an indicator strain for antimicrobial detection in muscle tissue (Explorer test), requiring a total work time of only 3 h based on *B. stearothermophilus* growth inhibition. To the best of our knowledge, our study is the first to use *B. stearothermophilus* as an indicator strain to detect carbapenemase activity. Furthermore, higher growth temperatures reduce potential issues from interference by mesophilic contaminants, making the application of *B. stearothermophilus* more promising in the clinical microbiology laboratory. The method uses a color change of the pH indicator based on acid production by the growth of the indicator bacteria. Other acid-producing carbapenemase-sensitive bacteria may also be used as indicator strains.

At the same time, as described in the results of use of mCIM detecting CR-non CPPA, even if the disk contains antibiotics, the test strain can still grow around the disk, thus interfering with the result of interpretation (Figure S2). However, using *B. stearothermophilus* as indicator strain, the colorimetric plates requiring the incubation of 60°C resulted in growth inhibition of mesophilic contaminants, which makes the interpretation of the results more clear.

A rapid carbapenem inactivation method (rCIM) was recently developed that could detect CPE within 3 h, but it required automated turbidity measurements, which may be a restriction for some clinical laboratories (21). Some researchers have attempted shorter incubation periods of the disk on the indicator strain (8 h), aiming to reduce incubation time of mCIM, but it is still time-consuming (22).

In summary, the CIM^B.S^ is a rapid colorimetric CRGB phenotypic detection method that has a sensitivity and specificity comparable with that of mCIM and detection time comparable with that of Blue-Carba. At the same time, CIM^B.S^ is a consistent and cost-effective assay, which can be adopted as a tool to detect CRGB on a routine basis in resource-limited regions. However, this test is qualitative, making it difficult for laboratory personnel to quantify uncertainty. The major limitation of this study is that the tested carbapenem-resistant Gram-negative bacteria only possessed several prevalent carbapenemases, in particular lacking the genotype like OXA-48, which is necessary to further study. In addition, we did not examine clinical samples of blood and urine, but these samples could be applicable because a carbapenem hydrolysis test for these sample types has been developed (22). Evaluation of the CIM^B.S^ assay to detect clinical samples is worthy of further study.

## Data Availability Statement

All datasets generated for this study are included in the article/Supplementary Material.

## Ethics Statement

This study uses clinical isolates were provided by the Huizhou First People's Hospital and The Third Affiliated Hospital of Sun Yat-sen University. SCAU Institutional Ethics Committee did not require the study to be reviewed or approved by an ethics committee because we are not involved in the isolation of bacteria.

## Author Contributions

JS, Y-HL, and X-PL designed the study. Z-HC, LJ, TT, LH, W-NN, and Z-XZ carried out the experiments. JS, M-GW, and X-RW analyzed the data. JS, Z-HC, and L-XF wrote the draft of the manuscript. All authors read and approved the final manuscript.

## Conflict of Interest

The authors declare that the research was conducted in the absence of any commercial or financial relationships that could be construed as a potential conflict of interest.

## References

[1] Lutgring JD Limbago BM. The problem of carbapenemase-producing-carbapenem-resistant-enterobacteriaceae detection. J Clin Microbiol. (2016) 54:529–34. 10.1128/JCM.02771-15 26739152 PMC4767976

[2] Zilberberg MD Nathanson BH Sulham K Fan W Shorr AF. Carbapenem resistance, inappropriate empiric treatment and outcomes among patients hospitalized with Enterobacteriaceae urinary tract infection, pneumonia and sepsis. BMC Infect Dis. (2017) 17:279. 10.1186/s12879-017-2383-z 28415969 PMC5393012

[3] Marchaim D Chopra T Pogue JM Perez F Hujer AM Rudin S. Outbreak of colistin-resistant, carbapenem-resistantklebsiella pneumoniaein metropolitan detroit, michigan. Antimicrob Agents Chemother. (2011) 55:593–9. 10.1128/AAC.01020-10 21115786 PMC3028794

[4] Nordmann P Naas T Poirel L. Global spread of carbapenemase-producing enterobacteriaceae. Emerg Infect Dis. (2011) 17:1791–8. 10.3201/eid1710.110655 22000347 PMC3310682

[5] Dortet L Poirel L Nordmann P. Worldwide dissemination of the NDM-type carbapenemases in Gram-negative bacteria. Biomed Res Int. (2014) 2014:249856. 10.1155/2014/249856 24790993 PMC3984790

[6] Roschanski N Hadziabdic S Borowiak M Malorny B Tenhagen BA Projahn M. Detection of VIM-1-producing enterobacter cloacae and salmonella enterica serovars infantis and goldcoast at a breeding pig farm in Germany in 2017 and their molecular relationship to former VIM-1-Producing S. infantis isolates in german livestock production. mSphere. (2019) 4:e00089–19. 10.1128/mSphere.00089-19 31189558 PMC6563352

[7] Nordmann P Poirel L. Strategies for identification of carbapenemase-producing Enterobacteriaceae. J Antimicrob Chemother. (2013) 68:487–9. 10.1093/jac/dks426 23104494

[8] Lutgring JD Zhu W de Man TJB Avillan JJ Anderson KF Lonsway DR. Phenotypic and genotypic characterization of enterobacteriaceae producing Oxacillinase-48-Like carbapenemases, United States. Emerg Infect Dis. (2018) 24:700–9. 10.3201/eid2404.171377 29553324 PMC5875285

[9] Hrabak J Chudackova E Papagiannitsis CC. Detection of carbapenemases in Enterobacteriaceae: a challenge for diagnostic microbiological laboratories. Clin Microbiol Infect. (2014) 20:839–53. 10.1111/1469-0691.12678 24813781

[10] Dortet L Tande D de Briel D Bernabeu S Lasserre C Gregorowicz G. MALDI-TOF for the rapid detection of carbapenemase-producing Enterobacteriaceae: comparison of the commercialized MBT STAR(R)-Carba IVD Kit with two in-house MALDI-TOF techniques and the RAPIDEC(R) CARBA NP. J Antimicrob Chemother. (2018) 73:3359–67. 10.1093/jac/dky209 29897463

[11] Boutal Hervé Naas T Devilliers K Oueslati S Dortet L Bernabeu S. Development and validation of a lateral flow immunoassay for rapid detection of NDM-producing enterobacteriaceae. J Clin Microbiol. (2017) 55:2018–29. 10.1128/JCM.00248-17 28404680 PMC5483903

[12] CLSI Performance Standards for Antimicrobial Susceptibility Testing: 21st Informational Supplement M100-S21. Wayne, PA: Clinical and Laboratory Standards Institute (2012).

[13] CLSI Performance Standards for Antimicrobial Susceptibility Testing; 27th Informational Supplement. CLSI M100-S27. Wayne, PA: Clinical and Laboratory Standards Institute (2017).

[14] Hussein AH Lisowska BK Leak DJ. The genus geobacillus and their biotechnological potential. Adv Appl Microbiol. (2015) 92:1–48. 10.1016/bs.aambs.2015.03.001 26003932

[15] Poirel L Walsh TR Cuvillier V Nordmann P. Multiplex PCR for detection of acquired carbapenemase genes. Diagn Microbiol Infect Dis. (2011) 70:119–23. 10.1016/j.diagmicrobio.2010.12.002 21398074

[16] CLSI. Performance Standards for Antimicrobial Susceptibility Testing CLSI M100-S25. Wayne, PA: Clinical and Laboratory Standards Institute (2015).

[17] Pires J Novais A Peixe L. Blue-carba, an easy biochemical test for detection of diverse carbapenemase producers directly from bacterial cultures. J Clin Microbiol. (2013) 51:4281–3. 10.1128/JCM.01634-13 24108615 PMC3838089

[18] Tijet N Boyd D Patel SN Mulvey MR Melano RG. Evaluation of the Carba NP test for rapid detection of carbapenemase-producing Enterobacteriaceae and Pseudomonas aeruginosa. Antimicrob Agents Chemother. (2013) 57:4578–80. 10.1128/AAC.00878-13 23817380 PMC3754310

[19] Wong MH Li Y Chan EW Chen S. Functional categorization of carbapenemase-mediated resistance by a combined genotyping and two-tiered Modified Hodge Test approach. Front Microbiol. (2015) 6:293. 10.3389/fmicb.2015.00293 25932021 PMC4399324

[20] Muntean MM Muntean AA Gauthier L Creton E Cotellon G Popa MI. Evaluation of the rapid carbapenem inactivation method (rCIM): a phenotypic screening test for carbapenemase-producing Enterobacteriaceae. J Antimicrob Chemother. (2018) 73:900–908. 10.1093/jac/dkx519 29351668

[21] Beresford RW Maley M. Reduced incubation time of the modified carbapenem inactivation test and performance of carbapenem inactivation in a set of carbapenemase-producing enterobacteriaceae with a high proportion of bla IMP isolates. J Clin Microbiol. (2019) 57:e01852-18. 10.1128/JCM.01852-18 30842234 PMC6595449

[22] Meier M Hamprecht A. Systematic comparison of four methods for the detection of carbapenemase-producing Enterobacterales (CPE) directly from blood cultures. J Clin Microbiol. (2019) 57:e00709–19. 10.1128/JCM.00709-19 31413083 PMC6813004

