# Supplementary material for: A Four-Hour Carbapenem Inactivation Method (CIMB.S) Using Bacillus stearothermophilus as Indicator Strain
Source: Front Med (Lausanne). 2020 Jul 31;7:364. doi: 10.3389/fmed.2020.00364 (PMC7411124; doi:10.3389/fmed.2020.00364)
Supplement: Supplementary file 1 [file Data_Sheet_1.PDF]

Supplementary Information for

Title: A four-hour Carbapenem Inactivation Method (CIM$^{B.S}$) using *Bacillus stearothermophilus* as indicator strain

Ze-Hua Cui[1,3], Ling Jia[1,3], Lu Han[1,3], Tian Tang[1,3], Zi-Xing Zhong[1,3], Liang-Xing Fang[1,3], Wei-Na Ni[1,3], Min-Ge Wang[1,3], Xi-Ran Wang[1,3], Ya-Hong Liu[1,2,3], Xiao-Ping Liao[1,2,3], Jian Sun[1,2,3*].

[1]National Risk Assessment Laboratory for Antimicrobial Resistance of Animal Original Bacteria, South China Agricultural University, Guangzhou, China.

[2] Guangdong Laboratory for Lingnan Modern Agriculture, Guangzhou, 510642, China.

[3]Laboratory of Veterinary Pharmacology, College of Veterinary Medicine, South China Agricultural University, Guangzhou 510642, P. R. China.

* Correspondence author:

Jian Sun.

E-mail: jiansun@scau.edu.cn.

Tel: +86-020-85285507; Fax: +86-020-85285507

Table S1. Source of test strains.

Species	n	Carbapenemase	specimens	Hospital
Test strains	134			
carbapenemase-producers	76			
E. coli	3	NDM-1	Fecal	A, B
E. coli	8	NDM-5	Fecal, Blood	B
E. coli	2	VIM-2	Fecal	B
E. coli	1	IMP-2	Fecal	B
K. pneumoniae	3	NDM-1	Blood	A
K. pneumoniae	3	NDM-5	Blood	A, B
K. pneumoniae	1	VIM-2	Blood	A
K. pneumoniae	1	IMP-2	Blood	A
K. pneumoniae	8	KPC-2	Blood, Urine	A, B
C. freundii	2	NDM-1	Urine	A
E. cloacae	3	NDM-1	Urine	A
E. cloacae	1	VIM-1	Urine	A
E. cloacae	3	IMP-2	Fecal	A, B
P. aeruginosa	9	NDM-5	Blood	A, B
P. aeruginosa	4	VIM-2	Blood	A, B
P. aeruginosa	5	IMP-2	Blood	B
A. baumannii	1	NDM-1	Blood	A
A. baumannii	17	OXA-23	Blood, Fecal	B
A. baumannii	1	KPC-2	Urine	A
Non-carbapenemase-producers	58			
E. coli	18	-	Fecal	A, B
K. pneumoniae	6	-	Fecal, Urine, Blood	A
C. freundii	8	-	Fecal	A
E. cloacae	4	-	Fecal	A, B
P. aeruginosa	17	-	Fecal, Blood	B
A. baumannii	5	-	Urine, Blood	B

A, The Third Affiliated Hospital of Sun Yat-sen University.

B, Huizhou First People's Hospital.

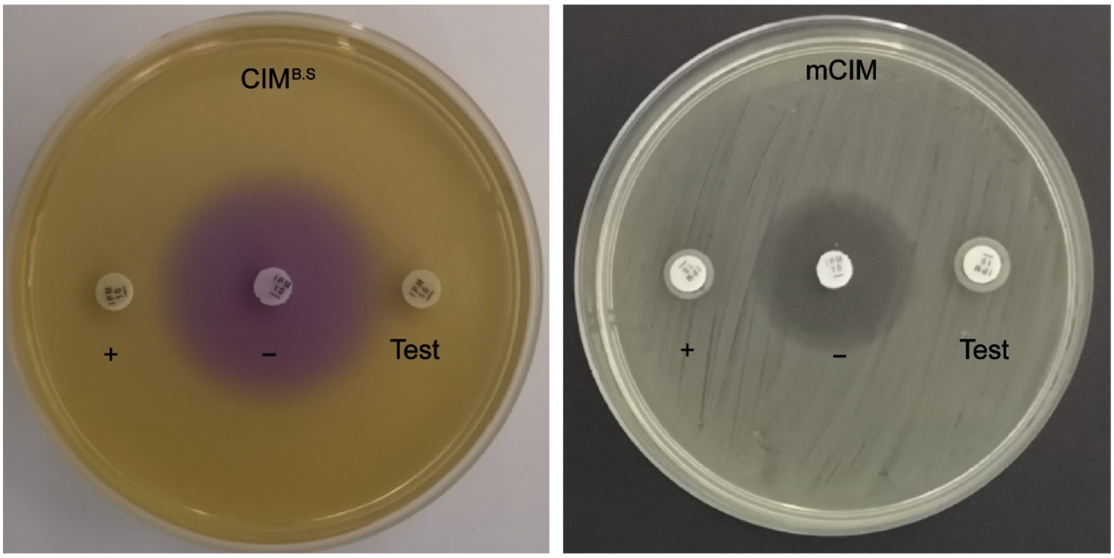

Figure S1. Representative results of the CIM$^{B.S}$ and mCIM. The re-incubation time of CIM$^{B.S}$ and mCIM were 3.5 h and 18 h, respectively.

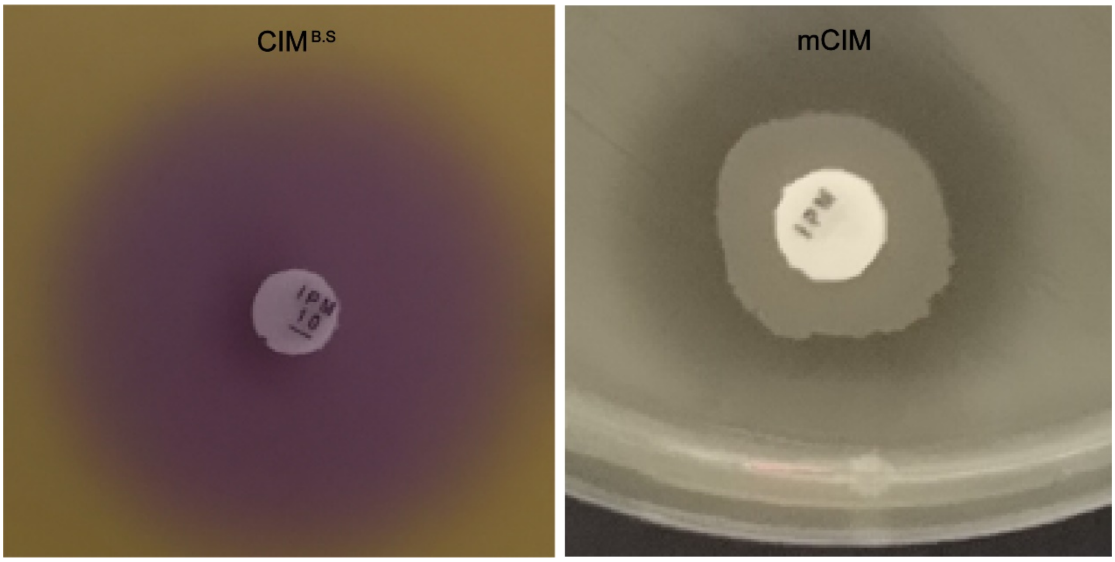

Figure S2. The results of CIM$^{B.S}$ and mCIM to detection one CR-non CPPA strain. The mCIM may interfere with the results, but CIM$^{B.S}$ does not.
